# Identifying Network Propagation Sources Using Advanced Centrality Measures

**DOI:** 10.3390/e27090948

**Published:** 2025-09-12

**Authors:** Damian Frąszczak

**Affiliations:** Institute of Information Systems, Faculty of Cybernetics, Military University of Technology, 00-908 Warsaw, Poland; damian.fraszczak@wat.edu.pl

**Keywords:** social network, propagation graph, propagation source identification, centrality measures

## Abstract

We live in a time dominated by interconnected networks surrounding us on all fronts. The emergence of social media platforms has driven the expansion of social networks, facilitating fast communication worldwide. Responses to content shared on these platforms can be seen as a propagation process, where information spreads through social networks. Analyzing propagation graphs presents a significant challenge in identifying sources, which is crucial in various fields. This includes detecting the origins of disinformation, identifying patient zero in an epidemic, and tracing the initial sources of viral trends or malware. Numerous studies have attempted to identify these sources using methods similar to centrality measures which assign a value indicating the likelihood of being a source. While centrality measures are a popular topic, with many new measures introduced each year, only a few have been explored in the context of source identification. This article explores a wide range of centrality measures in the context of source identification. The results help identify the most effective measures and pave the way for the development of more efficient detection techniques. Additionally, an analysis was conducted considering multiple hops in the propagation network, providing deeper insights into the impact of extended neighborhood structures on detection performance.

## 1. Introduction and Research Motivation

We live in an era where networks are ubiquitous, influencing many aspects of our daily lives. The emergence and widespread adoption of social media platforms have significantly accelerated the growth of social networks, allowing instant communication and content sharing across the globe. As information spreads quickly through these networks, understanding how it spreads is very important [1,2,3,4]. Reactions to content published on social media can be understood as a form of propagation, where information spreads through interconnected nodes in the network. The problem of identifying the source, often referred to as source identification, has recently gained more attention as a strategy for effectively controlling the spread of information [5,6,7].

Identifying the source of propagation in networks is a complex challenge with significant implications, such as tracing fake news, locating “patient zero” in disease outbreaks, and uncovering the origin of viral trends and malware. Despite its importance, source identification remains a challenging task [8,9]. Various methods, including Maximum Likelihood and Maximum A Posteriori estimators, Belief Propagation, Monte Carlo simulations, and additional approaches, have been developed to address this issue [5,6,8,10,11,12,13,14]. However, only a few centrality measures have been adequately explored in the context of source identification [15,16].

The issue of source detection is well-studied in the literature [6,11,12]. Works [15,16,17] present results based on well-known node centrality measures. Additionally, several studies introduce new metrics specifically designed for the source identification problem. For example, targeted betweenness centrality [18] is calculated within subgraphs identified using the Louvain method. Work [19] proposes the distance center, defined as the sum of the shortest paths from a node to all others, with the node with the smallest value considered the source. Ref. [20] introduces the unbiased betweenness centrality, computed as standard betweenness divided by the node degree.

Although various centrality-based approaches have been used for source detection [15,16,17,18,19,20,21], it should be emphasized that only a few popular ones have been widely applied. Some other techniques may have been proposed, but to the best of our knowledge, this is the first work to systematically evaluate as many as 25 different centrality measures in the context of source identification. The performance of these methods was compared with established rumor source detection techniques, such as RumorCenter [22], Netsleuth [23], and JordanCenter [24], across real and synthetic network structures using propagation simulations based on the SIR epidemiological model and the Independent Cascade model. The analysis employed traditional metrics, including the confusion matrix, precision, recall, F1 score, and source detection metrics such as average distance. The findings help identify effective centrality measures for source detection and guide future research by leveraging node characteristics considered by the best centrality measures.

In summary, the key aspects of the following research are as follows:

- A thorough analysis of advanced centrality measures for identifying the source of network propagation.
- For each simulation model, 96 unique propagation schemes were generated. Combining the two simulation models (SIR and IC) produced 192 propagation graphs. When these were paired with 25 tested methods, the total reached 4800 experiments.
- Evaluating source detection effectiveness through centrality measures, enhanced by including suspected sets within one- and two-hop neighborhoods, illustrates the potential of centrality for pinpointing areas suitable for more focused source detection.
- Experiments conducted on networks with various topologies confirmed the methods regarding both computational efficiency and detection performance.
- The study utilizes publicly available tools like NDLib [25] and NetCenLib [26], which confirm their practical value and encourage broader adoption in the research community.

This paper consists of six sections. The first section provides basic information and research motivation. The second section introduces social networks, the propagation process, source identification, and centrality measures. The third section outlines the techniques used in this study, while the fourth section details the simulation conditions. The fifth section presents the examinations conducted and the results obtained. Finally, the last section concludes the paper, summarizing key issues and suggesting future development directions.

## 2. Social Networks, Propagation, Source Identification, and Centrality Measures

The network is represented by an undirected graph G=(V,E), where nodes V (users/computers) are connected by edges E that denote relationships between them [6,10]. Spreading objects can initiate from one or more source nodes, which are known as sources v⊆G and whose positions significantly influence the speed of propagation [4,6,10,14]. Source nodes share information with neighboring nodes, encouraging their participation in the spread. Over time, as more nodes become involved, an infection/propagation $G_{I}$ graph forms. This graph, a subgraph G, consists of the infected nodes that have participated in the spread of information through their connections (edges). Source detection methods aim to identify these source nodes based on observed patterns. This paper applies the Maximum Likelihood (ML) approach, assuming no prior information about the source nodes is available. In such a case, all nodes are considered equally likely to be the origin of the spread. This reflects a realistic scenario in many real-world networks where the infection or information source is unknown. In this scenario $G_{I}$ plays a crucial role in the estimation process, as it contains indirect traces of the propagation dynamics. Even though no prior probabilities are assigned to individual nodes, the topology of this induced graph provides structural cues that source detection methods attempt to leverage to identify the most likely source. The corresponding optimization task consists of determining the nodes with the highest likelihood of being the origin of the spread:

(1) $$\hat{v}=\arg\underset{v\in G_{I}}{\max}P(G_{I}|v^{*}=v)$$

The $P(G_{I}|v^{*}=v)$ part is responsible for node evaluation and determining how probable it appears to be a source. One option for evaluating nodes is to use centrality measures. Centrality measures [2,27,28] evaluate specific characteristics of network vertices and determine which vertex is the most important based on a particular measure. The calculated values are usually normalized to a range of [0,1], making comparing them across different networks and identifying similarities easier. In essence, a centrality measure is a function $C:V\to \left[0,\;1\right]$ that provides these assessments. More details about the centrality measures used in this study will be provided further in this paper.

## 3. Research Background

To study the effectiveness of source identification techniques in network structures, it is essential to use propagation graph examples with labeled true sources. While the literature [29,30,31] provides some examples of such data, they are often limited by their focus on reactions to specific messages (e.g., tweets), their tree-like structure, small node counts, or a lack of information about the surrounding environment (e.g., the neighborhood) and finally propagation graph. To address these limitations, this research incorporates simulations using the SIR epidemiological model, a widely recognized approach for analyzing the effectiveness of such methods [4,6,8,10], as well as the opinion-based Independent Cascade (IC) model [32,33,34]. These models capture various aspects of disinformation spread on social networks. The SIR model reflects the general dynamics of information transmission and recovery, while the IC model simulates user-to-user influence based on individual activation [6,7,10].

Centrality measures and source identification algorithms have been validated on eight datasets, including real-world and synthetic networks. The dataset configurations are adapted from existing studies [10,35,36]. As summarized in Table 1, half of these datasets are derived from real-world networks, while the rest are synthetic, representing distinct properties of network structures. Real-world networks (Dolphin, Football, Facebook, and Social) reflect actual conditions to some extent, whereas synthetic ones (SC—scale-free and WS—small-world networks) help simulate various network properties. Moreover, the networks were selected to ensure the feasibility of loading and processing on the available hardware resources, while still reflecting diverse structural properties.

The propagation graphs were generated using a simulation-based approach with two diffusion models: the epidemiological SIR model and the IC model. Both models were applied under consistent conditions specific to each model, allowing for a fair comparison. The SIR model was adapted to emulate behaviors on real-world social media platforms, using an infection probability of 0.1 and a recovery probability of 0.05, as per [37], which reflects the realistic dynamics of COVID-19-related rumor propagation [37]. At the same time, the IC model adopted the simulation conditions presented in [34] with a transmission probability set to 0.4. Simulations were conducted across various networks, with 12 independent runs for each network–model combination, using initial infection sources set to 0.01%, 0.1%, 1%, and 10% of the nodes, giving 96 different propagation schemes per simulation model. In the case of smaller networks, when the calculated number of initially infected source nodes for different infection levels (e.g., 0.01% vs. 0.1%) resulted in the same value due to rounding, a fallback mechanism was applied. Specifically, the number of source nodes was set to the greater of 2 or the ceiling of the product of the total number of nodes and the fallback infection ratio. Each simulation aimed to reach at least 40% infection coverage. This value reached up to 80% in dense networks, while it ranged between 40% and 80% in sparser ones. In each run, source nodes were randomly selected with a uniform distribution, and the process ended once the target infection size was reached.

The problem of identifying sources of propagation can be formulated as a binary classification task, where nodes are classified as either source or non-source; thereby, standard evaluation metrics based on the confusion matrix are commonly applied to source detection problems. The confusion matrix and its meaning are presented in Table 2.

Based on values obtained in the confusion matrix, the following classification metrics are computed:

- Precision (PPV)

$$\mathrm{PPV}=\frac{TP}{TP+FP}$$

- Recall (TPR)

$$\mathrm{TPR}=\frac{TP}{TP+FN}$$

- F1

$$F1=\frac{2\cdot PPV\cdot TPR}{PPV+TPR}$$

Moreover, specific metrics for source detection evaluation are used, such as the following:

- Global Average Distance Error (GADE, hops)—the average shortest distance between a real source(s), v, and the estimated ones, $v^{*}$.

$$GADE=\frac{\sum_{\hat{v}}^{i=1}\underset{j\in v^{*}}{l(i,j)}}{\left|\hat{v}\right|}+\frac{\sum_{v^{*}\backslash \hat{v}}^{i=1}\underset{j\in \hat{v}}{l(i,j)}}{\left|\hat{v}\right|}$$

where l(i,j) is the shortest path length between nodes i and j.

- Average Detection Error (ADE)—the average ratio of the difference between the number of detected sources and the number of true sources.

$$\mathrm{ADE}=\frac{\sum_{N}^{i=1}ABS(\left|{\hat{v}}_{i}\right|-\left|{v^{*}}_{i}\right|)}{N}$$

where N is the number of experiments.

The research was conducted on a system with an Intel Core i7-10700 CPU running at 2.90 GHz, 64 GB of RAM, and an SSD, operating on the Linux Ubuntu platform. The analysis utilized the RPaSDT [38] package, which integrates libraries such as NetCenLib [26] for centrality measures and NSDLib [25] for source detection methods. This toolkit facilitated the preparation of rumor propagation experiments on various network topologies using NDLib [39] and enabled the analysis of source detection methods with widely recognized diffusion models. Although a Docker-based runtime environment was available, we utilized the standalone package designed for the Linux platform to reduce unnecessary load.

## 4. A Review of Centrality Metrics Used in the Research

In the study, implementations of available centrality measures from the NetCenLib [26] package were utilized, and the research employed only those measures applicable to undirected networks. A list of these measures is presented in Table 3.

- Algebraic centrality [40] measures the absolute and relative changes in a graph’s algebraic connectivity when a vertex is deleted.
- Average distance [41] centrality refers to the average length of the shortest paths from node u to all other nodes in the network. It represents the inverse of closeness centrality.
- Barycenter [42] quantifies a node’s centrality by the inverse of the total distance from that node to all others in the network. This measure identifies nodes centrally located in terms of overall network distance, reflecting their accessibility.
- Betweenness [43] measures the importance of a node by calculating the ratio of the shortest paths that pass through the node to the total number of shortest paths between all pairs of nodes in a network. It highlights nodes that frequently act as bridges along the shortest paths between other nodes, indicating their crucial role in the network’s connectivity.
- Closeness [44] measures how quickly a node can access all other nodes in a network, calculated as the inverse of the total distance from a node to all other nodes. This centrality metric often determines how rapidly information can spread from a given node to the entire network, highlighting strategically positioned nodes for efficient communication.
- ClusterRank [45] is a local ranking algorithm that evaluates a node’s influence by considering its direct connections, neighbors’ influence, and clustering coefficient. This approach enhances the evaluation by incorporating how closely interconnected a node’s neighborhood is. This can significantly improve the assessment of a node’s strategic importance in undirected networks compared to simple degree centrality or k-core decomposition methods.
- Coreness centrality [46], based on the k-shell indices of a node’s neighbors, is a powerful indicator of a node’s ability to disseminate information across a network. This metric evaluates a node’s connections to central or core network members, reflecting its potential influence more effectively.
- Current-flow betweenness [47] is the average amount of current flowing through a specific vertex, calculated across all possible pairs of source and target nodes within the network. This centrality measure averages the current flow for each node, indicating how much a node acts as a conduit for the flow between various pairs in the network. It is shown to be the same as random-walk betweenness [48,49].
- Current-flow closeness [47] transforms the traditional closeness index into a measure based on electrical current. This alternative approach calculates the distance between vertices in a network by assessing the difference in their electrical potentials. This method provides a distinctive perspective on node centrality, reflecting the ease with which current flows through different network parts. It is equivalent to information centrality [50].
- Decay [49,51] is a centrality measure that quantifies the importance of a vertex in a network based on its proximity to all other vertices, adjusted by a decay factor. Specifically, the decay centrality of a chosen vertex in a graph is calculated by weighting the closeness of this vertex to every other vertex by a decay factor, which diminishes the influence of distance.
- Degree measures the number of direct connections a node has in a network, indicating its importance based on how many neighbors it is directly linked to. A node with a higher degree of centrality is often more influential, as it interacts with other nodes directly [52].
- Diffusion degree [53] identifies the most influential nodes in a network by considering their direct connections and their ability to spread influence to neighbors. This measure captures the cumulative impact of a node and its neighbors during the diffusion process, reaching its maximum when all neighbors are successfully activated.
- Eigenvector [54] measures the influence of a node in a network by considering not only its direct connections but also the importance of the nodes it is connected to. It is computed using the principal eigenvector of the adjacency matrix, where the largest eigenvalue provides the desired centrality measure, ensuring that all entries in the eigenvector are positive.
- Geodesic K-path [27] measures the importance of a node by counting its neighbors within a geodesic path length of less than “k”.
- Harmonic [55] is a variation of closeness centrality that handles disconnected networks using the harmonic mean of distances, which performs better than the arithmetic mean when infinite distances are present. It calculates the importance of a node as the denormalized inverse of the harmonic mean of all distances to other nodes, making it more suitable for disconnected or sparse networks.
- Heatmap [56] combines local and global network information by comparing a node’s farness to the average farness of its neighboring nodes. A node with a smaller farness than its neighbors is considered more influential, as information is more likely to pass through it than through adjacent nodes. This makes heatmap centrality effective in identifying super-spreader nodes that control information flow within a network.
- Leverage [57] measures a node’s influence by comparing its degree to the degrees of its neighbors, averaging the differences. A node with negative leverage centrality is influenced by its neighbors, as they are more connected. In contrast, a node with positive leverage centrality has more influence over its neighbors, who have fewer connections.
- Lin [58] adjusts the concept of closeness by considering the average distance and the number of coreachable nodes. It is calculated by multiplying the inverse of the average distance by the square of the number of coreachable nodes, giving more importance to nodes that can reach a larger portion of the network. This modification ensures that nodes with larger coreachable sets are deemed more central, while nodes with no coreachable set have a centrality of 1 by definition.
- Load [59] measures the significance of a node by determining the fraction of all shortest paths in the network that go through it. This reflects the node’s ability to manage flow within the network. Unlike betweenness centrality, load centrality evenly distributes flow among neighboring nodes at the shortest distance to the target. This makes it especially valuable for analyzing flow structures operating below their capacity limits.
- MNC [60] evaluates the importance of a node by assessing the size of the largest connected group within its immediate neighbors, excluding the node itself. It measures how well-connected a node’s neighbors are, indicating the node’s influence based on the cohesion of its surrounding network.
- PageRank [61] evaluates the relative importance of nodes in a network by analyzing the number and quality of incoming links. It is based on a modified random walk, where there is a probability of jumping to any node, ensuring the scores are distributed more evenly across the network. Nodes with more links from highly ranked nodes are considered more important, making PageRank an effective way to measure influence within a network.
- Percolation [62] measures the importance of a node in spreading information or processes in dynamic networks, where nodes can transition between different states (e.g., infected or not). It calculates the proportion of shortest paths that pass through a node, considering its percolation state over time, making it useful for understanding the influence of spreading phenomena, such as diseases or information.
- Radiality [63] measures how close a node is to all other nodes in the network relative to the network’s diameter. A node with high radiality centrality is generally closer to other nodes, indicating a more central position, while a low radiality suggests the node is more peripheral in the network.
- Subgraph [64] measures how much a node participates in all the network subgraphs, accounting for all closed walks of various lengths that start and end at the node. It assigns a higher weight to shorter walks, meaning nodes involved in more local, tightly knit structures receive higher centrality. This centrality is calculated using the eigenvalues and eigenvectors of the network’s adjacency matrix.
- Topological [65] evaluates how much a node shares its neighbors with other nodes in the network. It calculates the ratio of shared neighbors between a node and its connected nodes, plus one if they have a direct connection, divided by the total number of neighbors of the node. Nodes with few or no neighbors receive a coefficient of zero, indicating minimal shared connections.

The above analysis highlights the key characteristics of the studied centrality measures, and the obtained results facilitate the identification of critical factors for detecting propagation sources.

## 5. Results and Discussion

**Figure 1:**
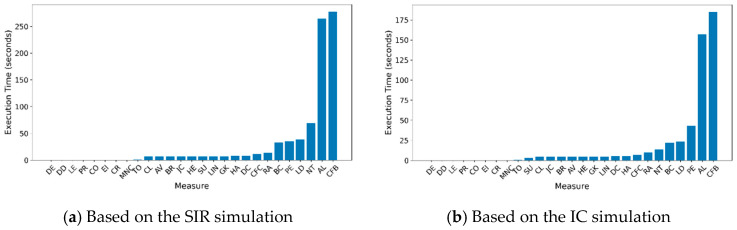
Average execution time per metric in different propagation models.

The research used simulations based on previously outlined conditions. The NetCenLib and NSDLib packages offer various centrality measures and techniques for identifying propagation sources. However, some methods, such as Hubbell centrality, are primarily designed for directed graphs, and others, like Rumor centrality, were too slow for this study, taking over 17 h to process the Social network under the lowest infection coverage. A maximum execution time of one hour was established, eliminating slower methods. Figure 1 illustrates the average execution times for the methods used, as summarized in Table 3. JordanCenter and NetSleuth are referred to as JC and NT, respectively. CFB and AL are among the slowest for both propagation methods, while NT, LD, PE, and BC are slower than average but faster than the slowest methods.

The methods used for the identification task were compared based on the F1 score and average error distance. The selected nodes matched the initial number of sources, focusing on those with the highest metric values.

**Figure 2:**
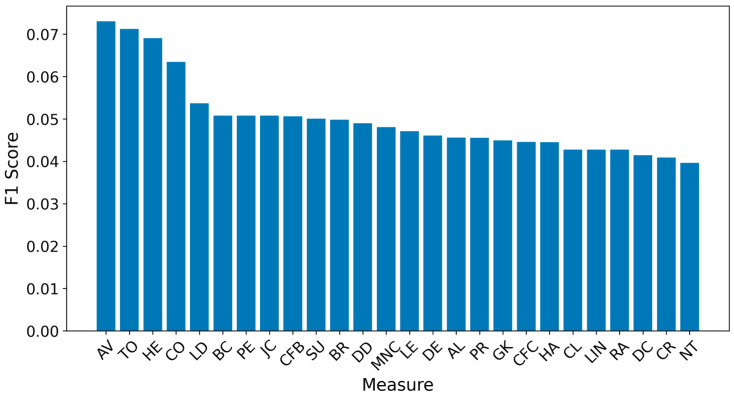
Global F1 score per measure.

**Figure 3:**
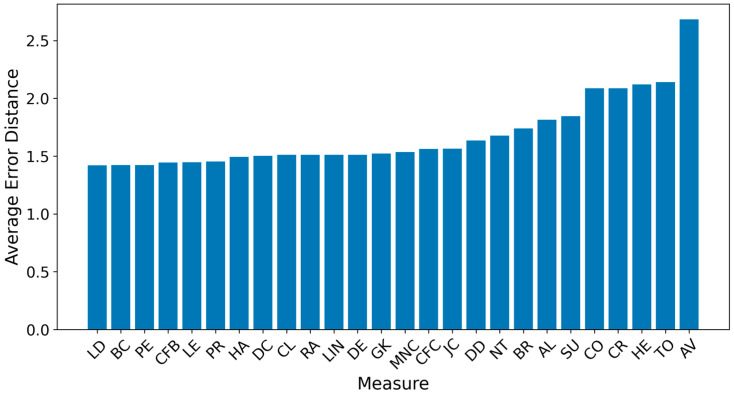
Global average distance per measure.

Figure 2 and Figure 3 present the overall results for propagation graphs generated by both simulation models, with F1 scores sorted in descending order and average distance errors in ascending order. Although the AV technique produced the best average F1 score, the small difference (0.03) makes it difficult to identify a clear best technique.

The average error distance graph revealed that LD, BC, PE, CFB, LE, PR, HA, DC, CL, RA, LIN, DE, GM, MNC, CFV, and JC performed the best, with distances of about 1.5 hops from the source, while AV was the worst, exceeding 2.5 hops. Notably, AV had the highest F1 score but the worst average distance. This can be explained by its tendency to correctly identify some sources while frequently choosing central nodes far from the true origins.

**Figure 4:**
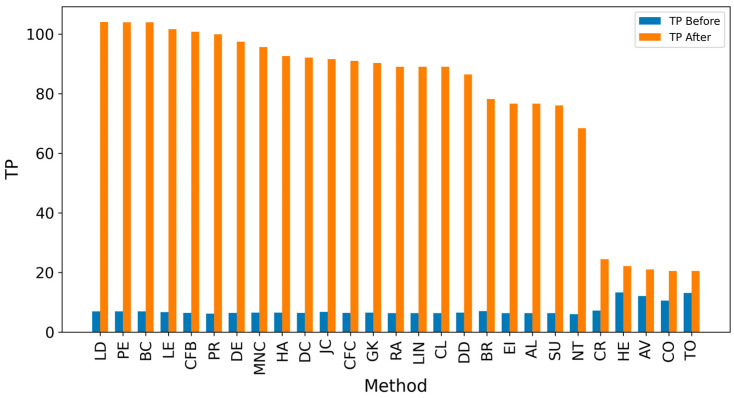
Global average TP score before and after expanding detected nodes by one hop per method.

**Figure 5:**
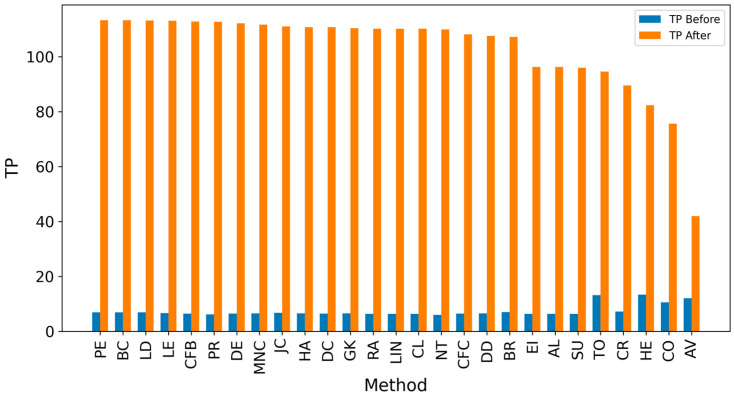
Global average TP score before and after expanding detected nodes by two hops per method.

**Figure 6:**
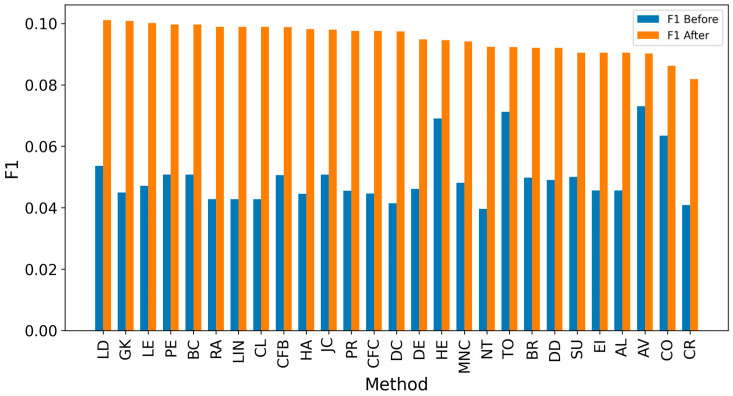
Global average F1 score before and after expanding detected nodes by one hop per method.

**Figure 7:**
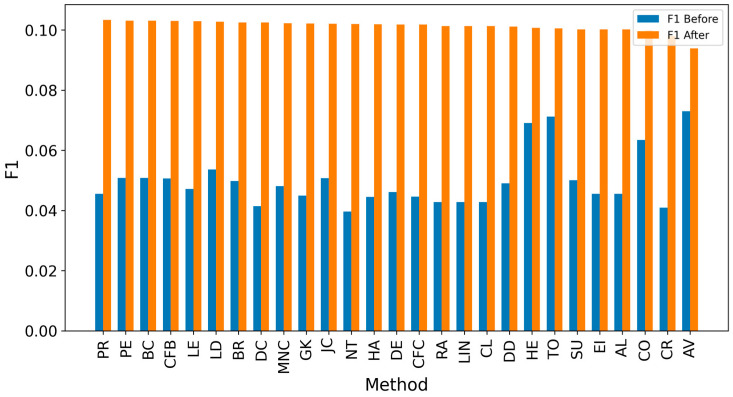
Global average F1 score before and after expanding detected nodes by two hops per method.

**Figure 8:**
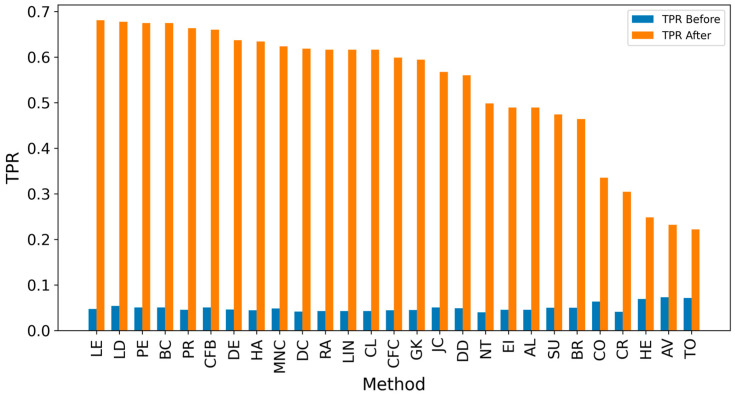
Global average TPR score before and after expanding detected nodes by one hop per method.

**Figure 9:**
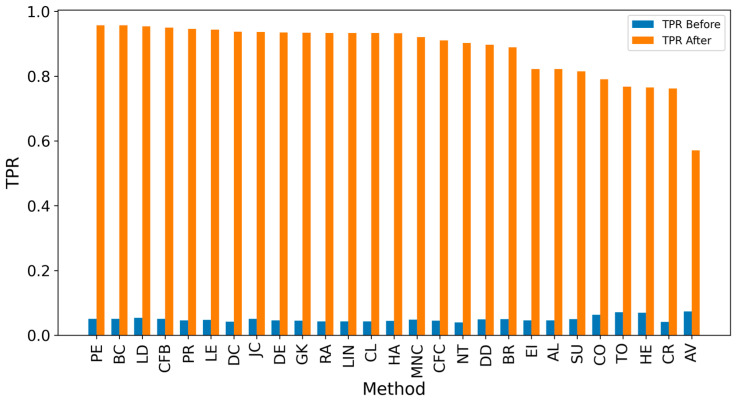
Global average TPR score before and after expanding detected nodes by two hops per method.

**Figure 10:**
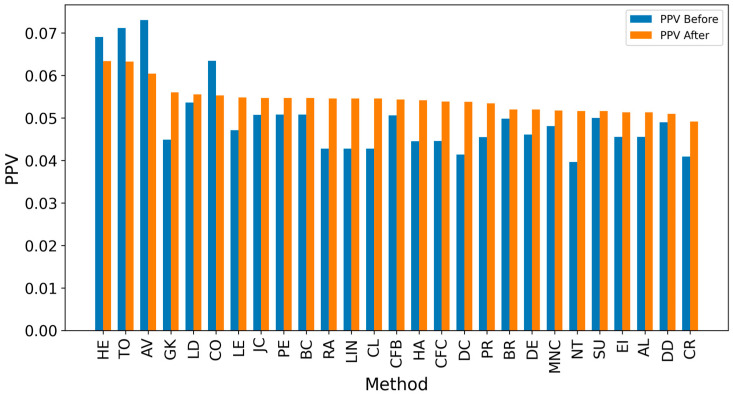
Global average PPV score before and after expanding detected nodes by one hop per method.

**Figure 11:**
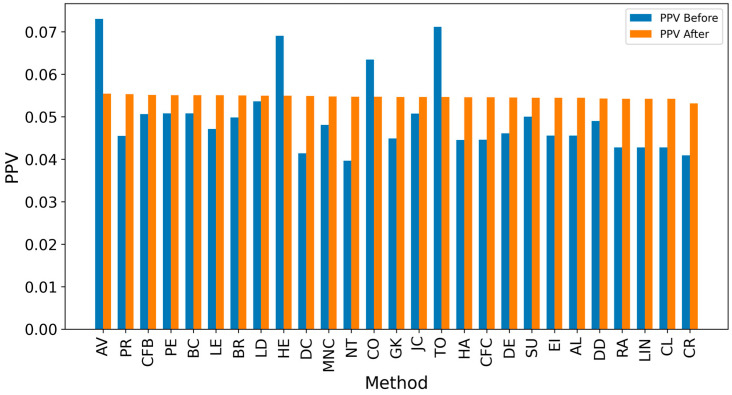
Global PPV score before and after expanding detected nodes by two hops per method.

Overall, achieving high accuracy in detecting real sources is a complex task, making it challenging to achieve high F1 scores. Since the average distance error is critical for evaluation, extending the detected sources to include neighbors could enhance results [8,10,12]. It can be observed that expanding the identified source nodes by including their immediate neighbors (one hop: Figures 4, 6, 8 and 10) or even their neighbors’ neighbors (two hops: Figures 5, 7, 9 and 11) improves the accuracy of detecting the real source nodes. The results reported in the paper are based on all experiment results with both propagation models (IC and SIR), while detailed results for specific cases are presented in the Appendix A and Appendix B.

In the TP measure (Figure 4 and Figure 5), more source nodes have been correctly identified due to a larger suspected set and increased hops. This is also reflected in the F1 scores (Figure 6 and Figure 7), which are higher than the original ones, with two-hop neighbors yielding similar results. However, there appears to be a limit around an F1 score of 0.11. The TPR (Figure 8 and Figure 9) metric shows significant improvement for both one-hop and two-hop nodes, and the PPV results (Figure 10 and Figure 11) decline when including two hops due to more false positives. The results suggest that using one-hop neighbors is optimal. Future improvements could go beyond the current practice of either selecting all identified nodes or only the one with the highest score [10,11,12]. Possible extensions include choosing the top-X nodes with similar or close obtained values, or even adopting approaches like [15], where an extra measure is used to identify crucial nodes. However, this optimization is beyond the scope of this paper.

Table 4 contains two subtables: comparing performance before (Table 4a) and after extending the analysis by one hop (Table 4b). The values are sorted by True Positive Rate (TPR), as this metric is critical in identifying as many sources as possible, which is essential in the propagation context. After the one-hop extension, there is a significant increase in TPR and True Positives (TP), with methods like LE and LD achieving TPR values of ~0.68, compared to ~0.05 in the initial setup. F1 scores also improved, with LD increasing from 0.05 to 0.101, but the differences in F1 are less pronounced than the gains in TPR. Interestingly, expanding the analysis led to changes in the rankings of methods, with LE becoming the top performer in TPR, while CFC was ranked higher in the original configuration. The best methods for TPR metrics are CFB and LD, and they leverage features related to the shortest paths passing through a node, highlighting that this characteristic is pivotal in identifying propagation sources.

F1 scores also improved, with LD increasing from 0.054 to 0.101, but the differences in F1 are less pronounced compared to the gains in TPR. Expanding the analysis led to changes in the ranking of methods, with LE and LD emerging as top performers in TPR, while AV was ranked higher in the original configuration. Notably, methods with a TPR above 0.65 consistently achieved relatively high F1 scores, demonstrating that better coverage correlates with a balanced precision and recall.

These results highlight that relying on a single centrality-based method may be insufficient for comprehensive source detection. The inclusion of one-hop extensions significantly improves detection but also introduces the possibility of refining the approach further. For instance, excluding boundary nodes or those with minimal likelihood of being sources could reduce false positives and improve precision. A key finding of this study is that features related to shortest paths play a crucial role in source identification. Future research should focus on integrating these extensions with selective node filtering to enhance both F1 scores and overall detection robustness.

The analysis of individual method evaluations in SIR and IC-based simulations, presented in Appendix A and Appendix B, respectively, led to interesting conclusions. Notably, the methods achieved higher metric values in the SIR model—for example, the highest F1 score was 0.16 compared to 0.105, and the TPR was 0.78 compared to 0.65. Moreover, the results show that centrality measures used for source identification yield different outcomes depending on the simulation model and network topology. It concludes that the choice of propagation model has a significant influence on the effectiveness of detection methods, underscoring the need to tailor techniques to specific spreading dynamics or network topologies.

**Figure 12:**
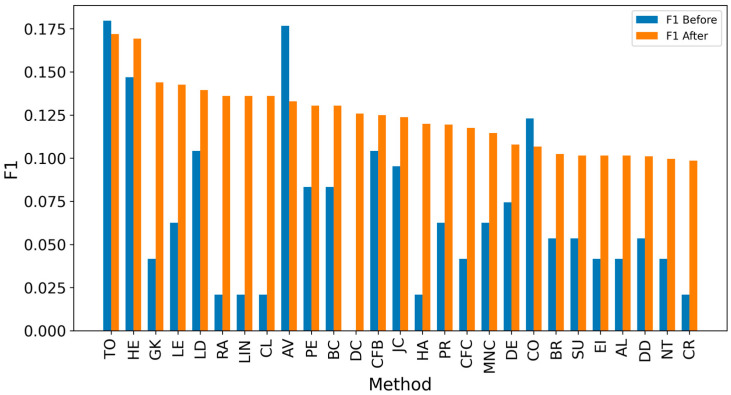
Global average F1 score before and after expanding detected nodes by one hop for the Dolphin network.

**Figure 13:**
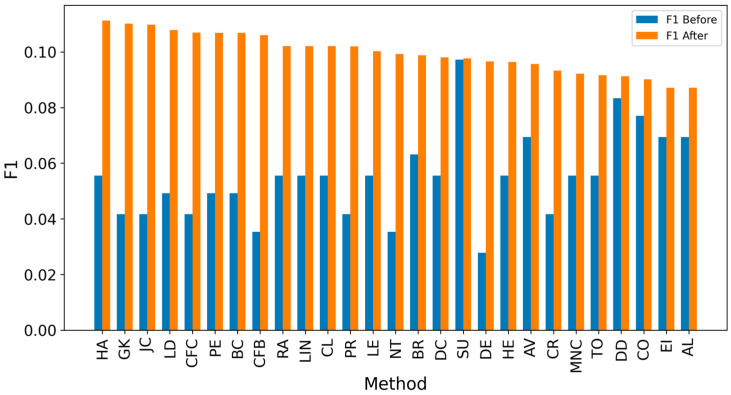
Global average F1 score before and after expanding detected nodes by one hop for the Football network.

**Figure 14:**
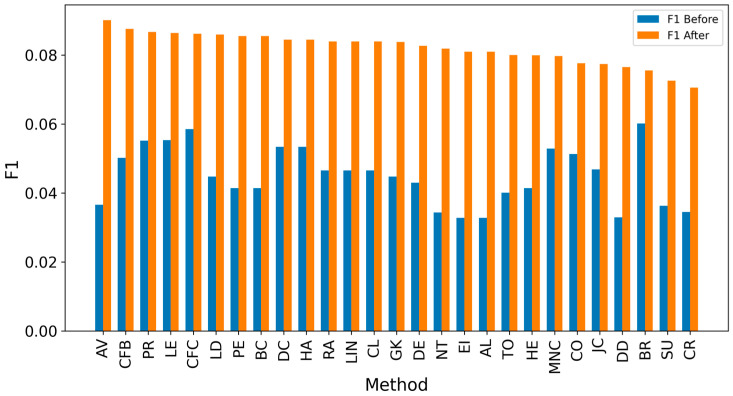
Global average F1 score before and after expanding detected nodes by one hop for the SW-1 network.

**Figure 15:**
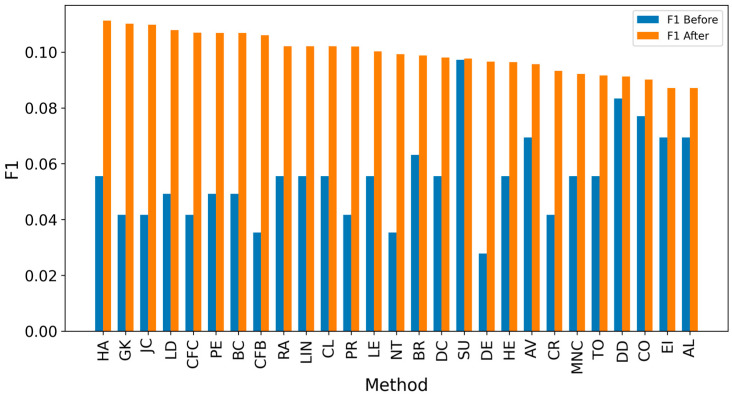
Global average F1 score before and after expanding detected nodes by one hop for the SW-2 network.

**Figure 16:**
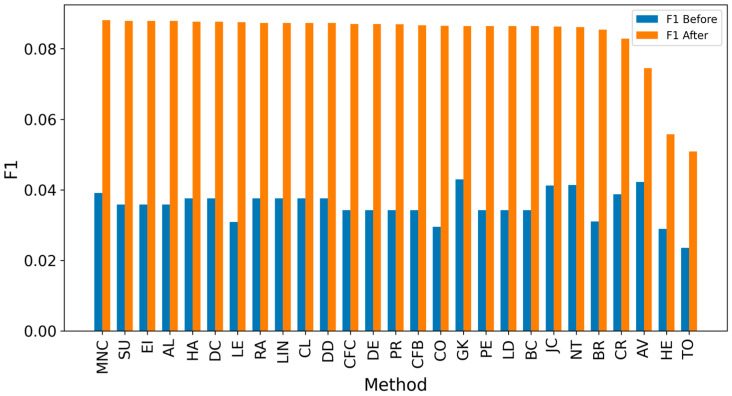
Global average F1 score before and after expanding detected nodes by one hop for the SF-1 network.

**Figure 17:**
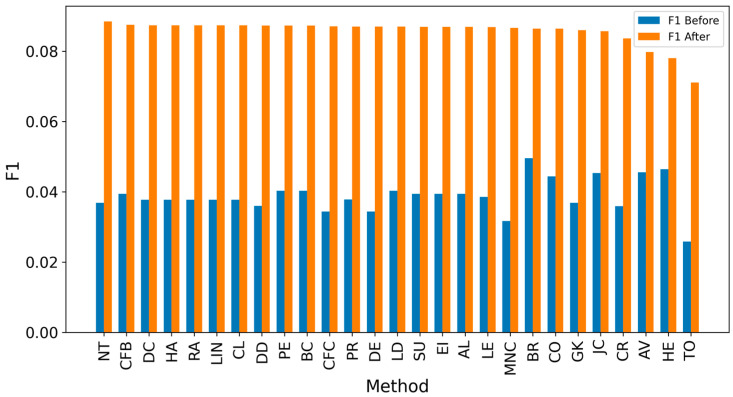
Global average F1 score before and after expanding detected nodes by one hop for the SF-2 network.

**Figure 18:**
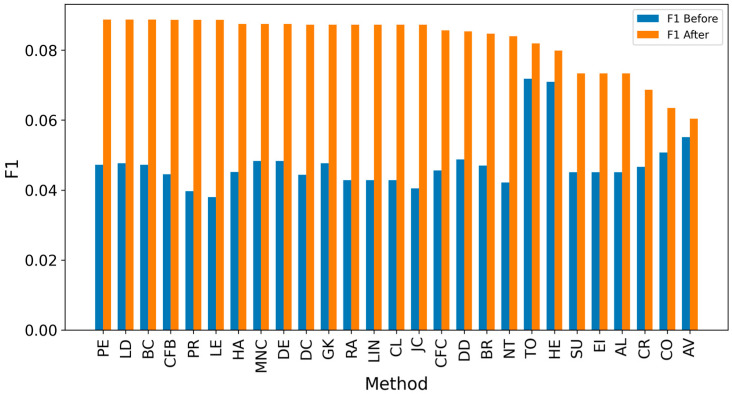
Global average F1 score before and after expanding detected nodes by one hop for the Facebook network.

**Figure 19:**
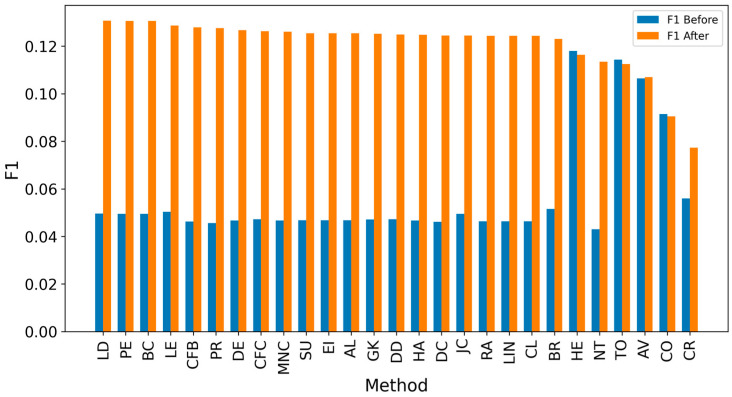
Global average F1 score before and after expanding detected nodes by one hop for the Social network.

The analysis of results by network type shows that in smaller networks, such as Dolphin (Figure 12) and Football (Figure 13), F1 scores exceed 0.175 due to the smaller number of source nodes. Similar results are observed in small-world synthetic networks (Figure 14 and Figure 15), where short distances between nodes facilitate easier tracking of information sources. In contrast, scale-free networks (Figure 16 and Figure 17) exhibit lower scores, often below 0.08, without expanding the set of suspected nodes. Larger social networks like Facebook (Figure 18) and Social (Figure 19) show a similar trend. However, expanding the suspected node set can double the scores in many cases. The better performance of small-world networks stems from shorter distances between nodes, allowing quicker identification of information sources. In scale-free networks, the presence of highly connected nodes (hubs) complicates source identification, leading to lower F1 scores. Interestingly, adding extra connections in small-world networks does not always improve outcomes, with initial source nodes often achieving higher F1 scores than extended ones. Real-world networks often display characteristics of both small-world and scale-free types. Moreover, denser networks, with more connections between nodes, improve source identification accuracy, as evidenced by lower average hop errors across methods in denser setups compared to sparser ones. This trend underscores the universal benefit of network density in improving source identification effectiveness.

## 6. Conclusions

This paper examines the utilization of various centrality measures to identify propagation sources in networks. The research was conducted on both real-world and generated social networks, with the propagation process simulated using two propagation models: the SIR and IC models. Each simulation began with randomly chosen source nodes, leading to a total of 96 propagation simulations for each network type and propagation model. Combining the two models (SIR and IC), this resulted in 192 propagation graphs, which, when evaluated with 25 different methods, summed up to a total of 4800 experiments. These simulations provided the propagation graphs needed to assess the various techniques used for source identification.

The analysis demonstrated that a diverse set of state-of-the-art centrality measures yielded better results for source identification than baseline methods specifically designed for this purpose, such as NETSLEUTH and the Jordan Center. This finding suggests that general centrality measures may be more effective in certain contexts than specialized methods. However, some more complex approaches, like RumorCenter, could not complete all tests within the one-hour time limit per propagation graph due to their computational complexity. This limitation is significant for real-world applications, where timely network analysis is crucial.

An extended analysis that considered multiple hops in the propagation network revealed a notable improvement in source identification performance. The True Positive Rate (TPR) significantly increased, especially for centrality measures based on shortest paths, such as LD and CFB, which proved to be the most effective techniques. These results underscore the importance of incorporating extended neighborhood structures in source detection. Furthermore, the correlation between high TPR and balanced F1 scores implies that methods leveraging shortest-path-related features are particularly well-suited for this task. This finding opens avenues for future enhancements in detection techniques, such as filtering out boundary nodes or nodes with a low likelihood of being sources, to enhance the results. Interestingly, the methods achieved varying results depending on the propagation graphs generated by different diffusion models. This leads to the conclusion that source detection methods should be selected according to the expected propagation dynamics. Although the overall F1 scores achieved were not entirely satisfactory, the study successfully identified the most effective techniques, providing a foundation for future improvements in source detection accuracy.

One potential direction for future work involves dividing the network into smaller subgroups centered around key propagation hubs, allowing for localized source identification. This approach, often referred to as propagation outbreak detection [14], can be resolved by community detection methods like Louvain, Leiden, or BLOCD [66,67,68]. By narrowing the detection scope to smaller, structurally coherent regions, it may be possible to improve accuracy while reducing computational complexity [69]. Another approach to leverage the findings is to apply effective centrality measures to identify candidate nodes within a one-hop neighborhood of the infected region. These nodes can serve as initial indicators of potential source locations. In the next step, a more detailed analysis—such as collecting timestamp data or applying computationally intensive methods—can focus only on this reduced area. This two-stage process can significantly lower computational costs while maintaining or even improving detection accuracy. Finally, the flexibility and openness of the developed tools, such as NetCenLib and NSDLib, provide a solid foundation for further experimentation and extension, using new centrality methods like Biharmonic distance [70] and others [49]. These libraries can be expanded with new detection algorithms, alternative centrality metrics, or domain-specific heuristics to support both academic research and practical applications in network monitoring and rumor source containment.

## Figures and Tables

**Table 1:** The networks and their analysis used in the study.

Network	Nodes	Edges	Density	Assortativity	Avg. Clustering Coefficient	Degree (min/avg./max)
Dolphin	62	159	0.0841	−0.0436	0.259	1/5.13/12
Football	115	613	0.0935	0.1624	0.4032	7/10/12
SF-1	500	2475	0.0198	−0.0966	0.0659	5/9.9/69
SW-1	500	2500	0.0200	−0.0244	0.1640	5/10.0/16
SF-2	1000	4975	0.0100	−0.0613	0.0423	5/9.95/126
SW-2	1000	5000	0.0100	−0.0061	0.1478	5/10.0/16
Facebook	4039	88,234	0.0108	0.0636	0.6055	1/44/1045
Social	12,600	671,000	0.0008	−0.1219	0.2275	1/10/8700

**Table 2:** Confusion matrix explanation.

	Predicted Positive	Predicted Negative
Actually positive	TP (true positive)	FN (false negative)
Actually negative	FP (false positive)	TN (true negative)

**Table 3:** List of the centrality measures used in this research.

Centrality Measure		
Algebraic [AL]	Decay [DC]	Load [LD]
Average distance [AV]	Degree [DE]	MNC [MNC]
Barycenter [BR]	Diffusion degree [DD]	PageRank [PR]
Betweenness [BC]	Eigenvector [EI]	Percolation [PE]
Closeness [CL]	Geodestic k path [GK]	Radiality [RA]
Cluster rank [CR]	Harmonic [HA]	Subgraph [SU]
Coreness [CO]	Heatmap [HE]	Topological [TO]
Current Ffow betweenness (random walk betweenness) [CFB]	Leverage [LE]	
Current flow closeness (information centrality) [CFC]	Lin [LIN]	

**Table 4:** Performance metrics of propagation source detection methods for all experiments: comparison of average TP, F1, TPR, and PPV. (a) Directly identified sources. (b) Sources extended by one hop.

(a)				
	TP	F1	TPR	PPV
AV	12.083	0.073	0.073	0.073
TO	13.146	0.071	0.071	0.071
HE	13.333	0.069	0.069	0.069
CO	10.510	0.063	0.063	0.063
LD	6.927	0.054	0.054	0.054
PE	6.906	0.051	0.051	0.051
BC	6,906	0.051	0.051	0.051
JC	6.760	0.051	0.051	0.051
CFB	6.406	0.051	0.051	0.051
SU	6.313	0.050	0.050	0.050
BR	7.042	0.050	0.050	0.050
DD	6.521	0.049	0.049	0.049
MNC	6.500	0.048	0.048	0.048
LE	6.667	0.047	0.047	0.047
DE	6.469	0.046	0.046	0.046
AL	6.333	0.046	0.046	0.046
EI	6.333	0.046	0.046	0.046
PR	6.167	0.046	0.046	0.046
GK	6.552	0.045	0.045	0.045
CFC	6.427	0.045	0.045	0.045
HA	6.500	0.045	0.045	0.045
CL	6.365	0.043	0.043	0.043
LIN	6.365	0.043	0.043	0.043
RA	6.365	0.043	0.043	0.043
DC	6.406	0.041	0.041	0.041
CR	7.219	0.041	0.041	0.041
NT	6.021	0.040	0.040	0.040
(**b**)				
	**TP**	**F1**	**TPR**	**PPV**
LD	103.969	0.101	0.677	0.056
GK	90.250	0.101	0.594	0.056
LE	101.635	0.100	0.681	0.055
PE	103.958	0.100	0.675	0.055
BC	103.958	0.100	0.675	0.055
LIN	88.948	0.099	0.616	0.055
RA	88.948	0.099	0.616	0.055
CL	88.948	0.099	0.616	0.055
CFB	100.760	0.099	0.660	0.054
HA	92.604	0.098	0.634	0.054
JC	91.521	0.098	0.568	0.055
PR	99.906	0.098	0.664	0.053
CFC	90.948	0.098	0.599	0.054
DC	92.063	0.097	0.619	0.054
DE	97.427	0.095	0.637	0.052
HE	22.146	0.095	0.248	0.063
MNC	95.573	0.094	0.623	0.052
NT	68.396	0.092	0.498	0.052
TO	20.500	0.092	0.222	0.063
BR	78.146	0.092	0.464	0.052
DD	86.375	0.092	0.560	0.051
SU	76.010	0.090	0.474	0.052
AL	76.656	0.090	0.490	0.051
EI	76.656	0.090	0.490	0.051
AV	21.031	0.090	0.232	0.060
CO	20.531	0.086	0.335	0.055
CR	24.458	0.082	0.305	0.049

**Figure A1:**
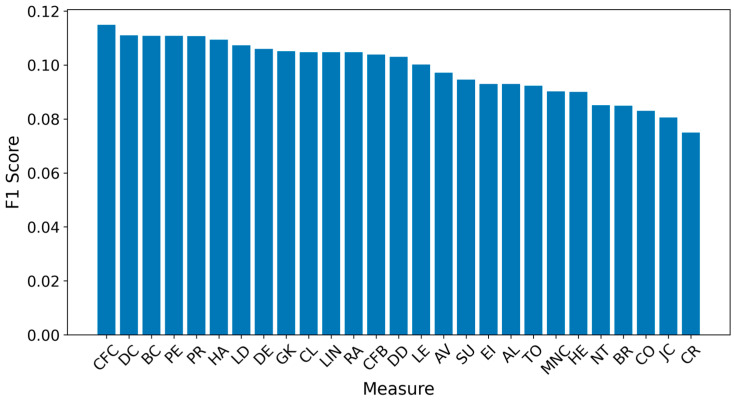
Global F1 score per measure in the SIR-based simulation.

**Figure A2:**
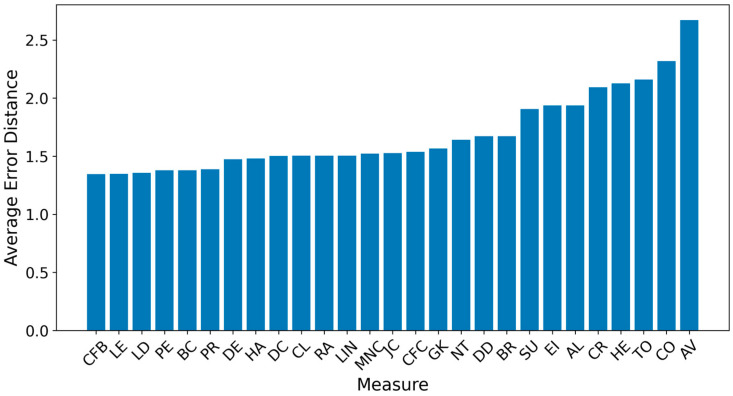
Global average distance per measure in the SIR-based simulation.

**Figure A3:**
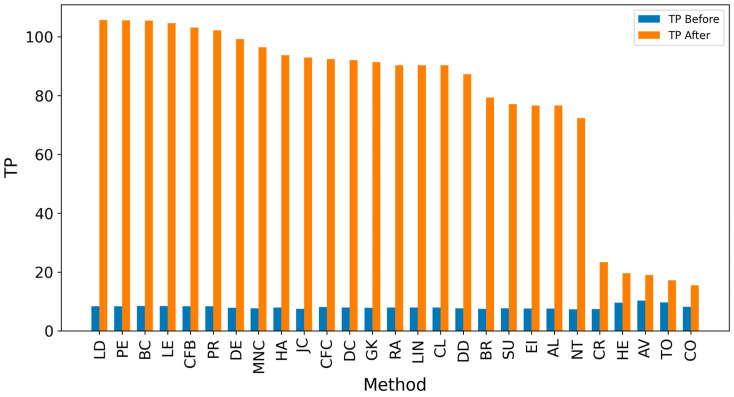
Average TP score before and after expanding detected nodes by one hop per method in the SIR-based simulation.

**Figure A4:**
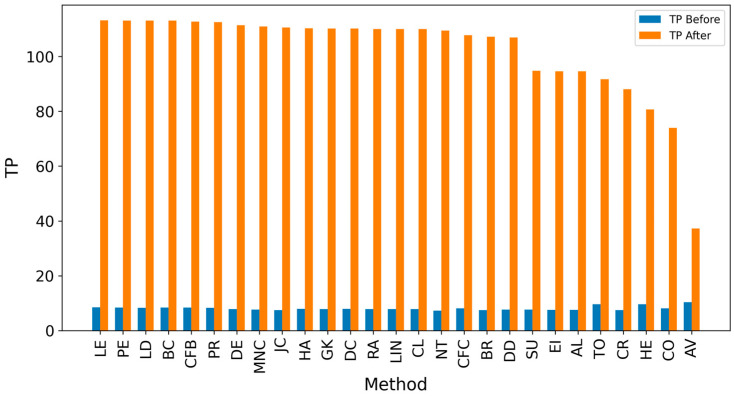
Average TP score before and after expanding detected nodes by two hops per method in the SIR-based simulation.

**Figure A5:**
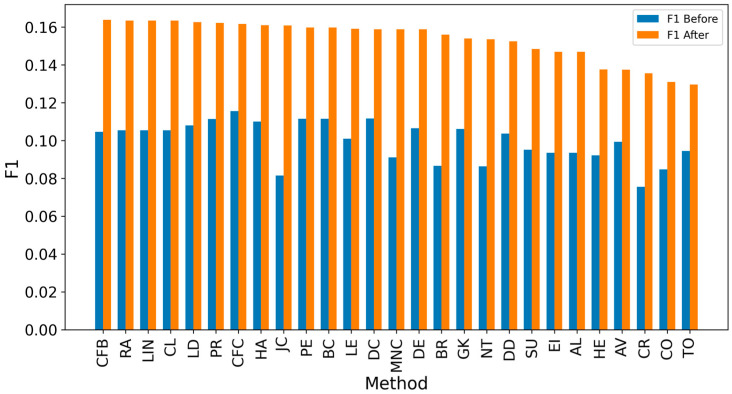
Average F1 score before and after expanding detected nodes by one hop per method in the SIR-based simulation.

**Figure A6:**
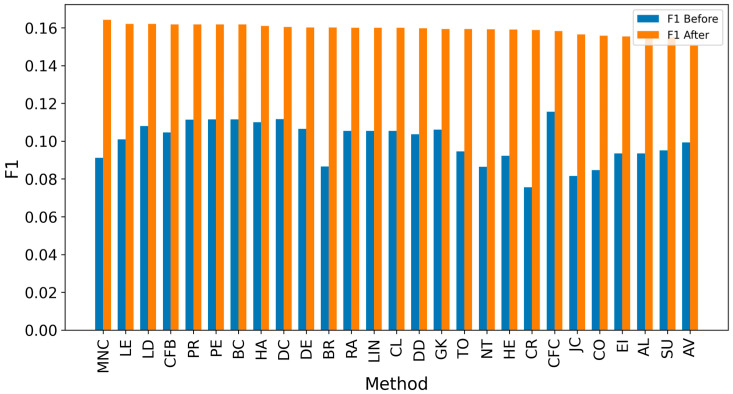
Average F1 score before and after expanding detected nodes by two hops per method in the SIR-based simulation.

**Figure A7:**
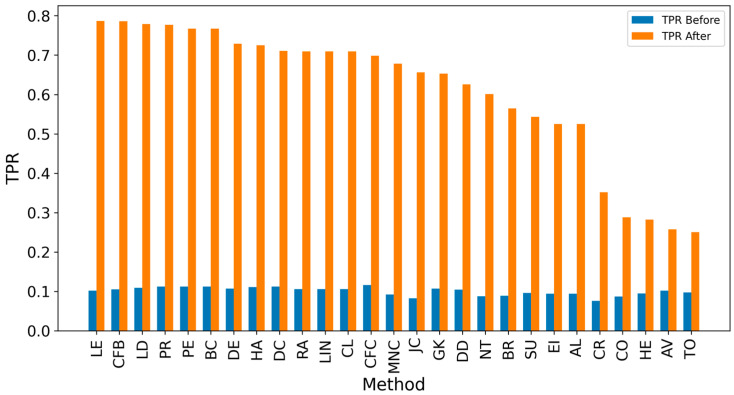
Average TPR score before and after expanding detected nodes by one hop per method in the SIR-based simulation.

**Figure A8:**
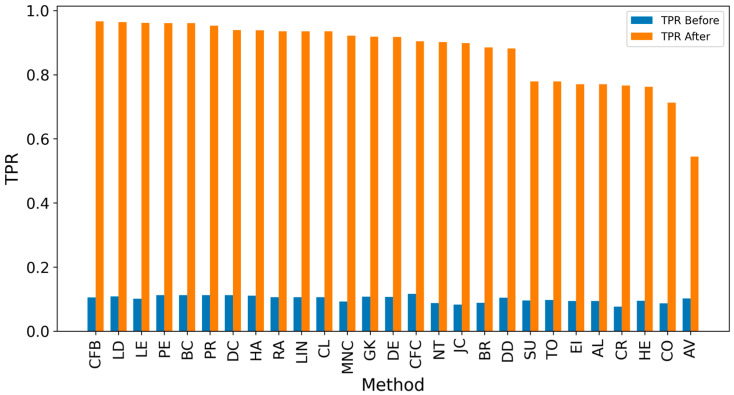
Average TPR score before and after expanding detected nodes by two hops per method in the SIR-based simulation.

**Figure A9:**
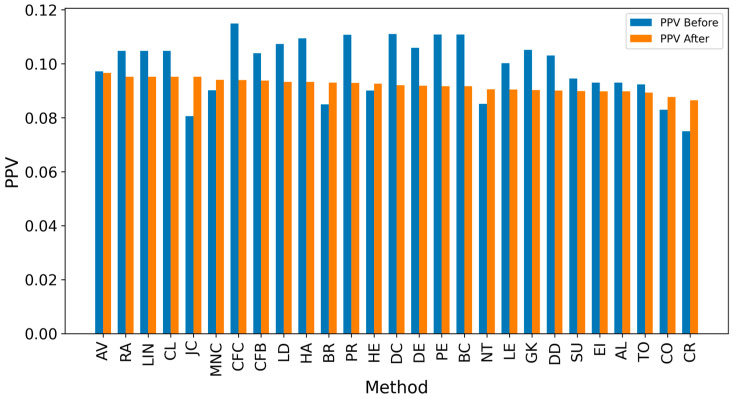
Average PPV score before and after expanding detected nodes by one hop per method in the SIR-based simulation.

**Figure A10:**
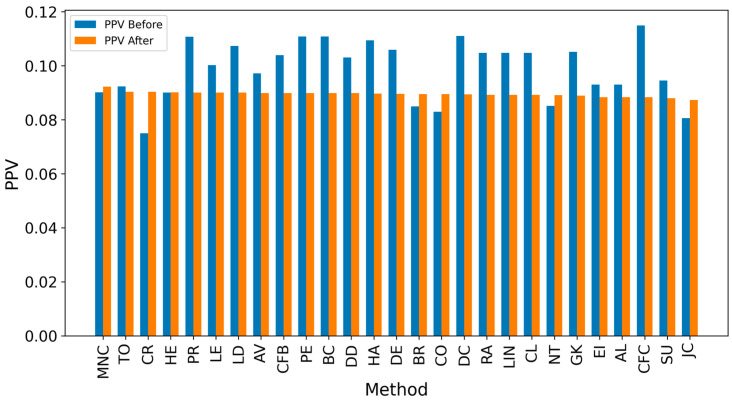
Average PPV score before and after expanding detected nodes by two hops per method in the SIR-based simulation.

**Figure A11:**
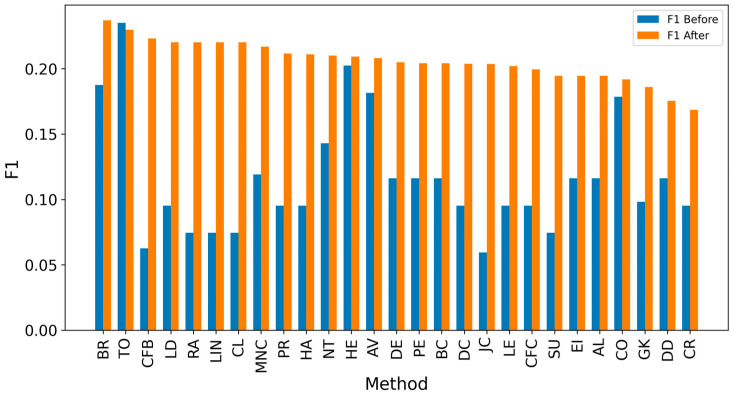
Average F1 score before and after expanding detected nodes by one hop for the Dolphin network in the SIR-based simulation.

**Figure A12:**
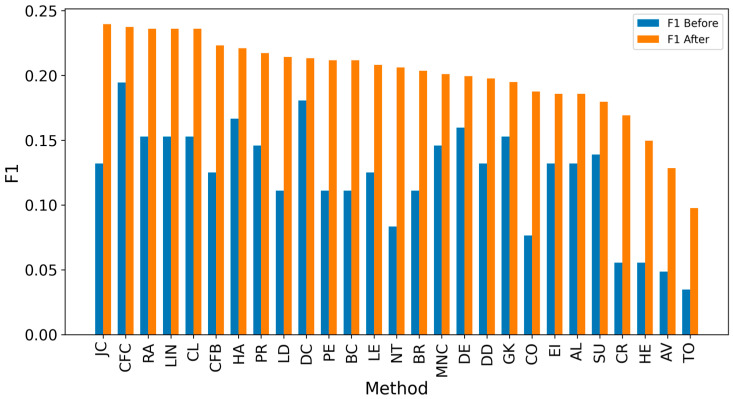
Average F1 score before and after expanding detected nodes by one hop for the Football network in the SIR-based simulation.

**Figure A13:**
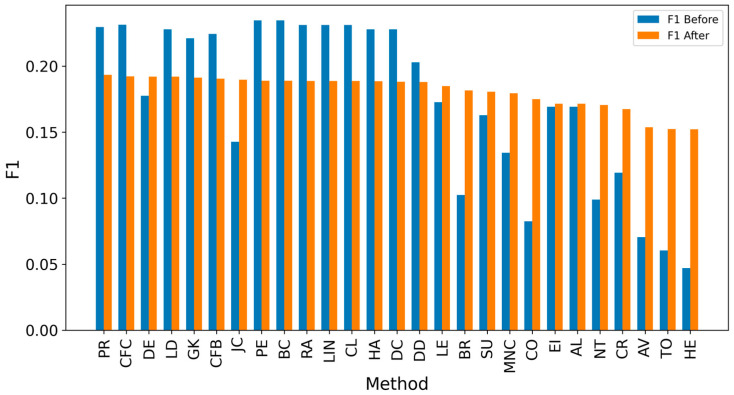
Average F1 score before and after expanding detected nodes by one hop for the SW-1 network in the SIR-based simulation.

**Figure A14:**
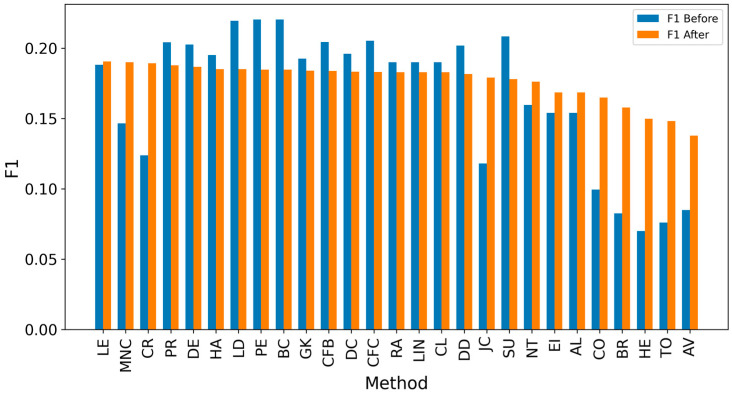
Average F1 score before and after expanding detected nodes by one hop for the SW-2 network in the SIR-based simulation.

**Figure A15:**
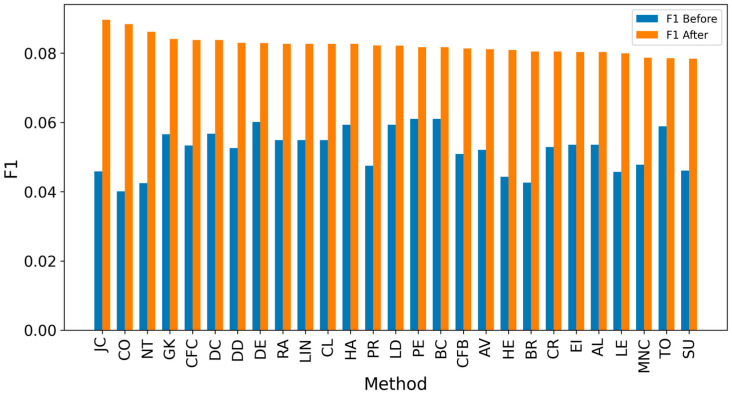
Average F1 score before and after expanding detected nodes by one hop for the SF-1 network in the SIR-based simulation.

**Figure A16:**
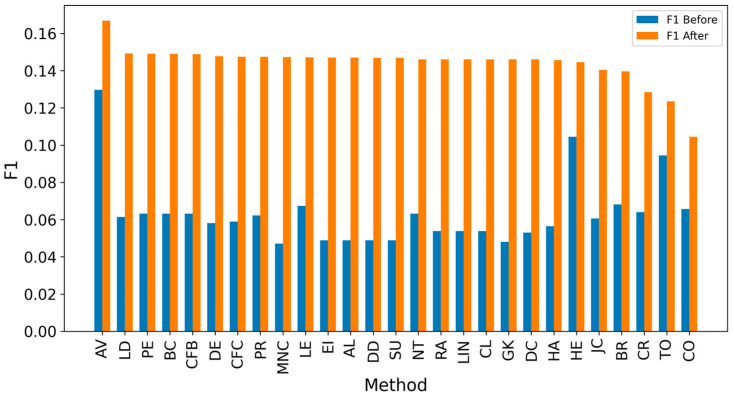
Average F1 score before and after expanding detected nodes by one hop for the SF-2 network in the SIR-based simulation.

**Figure A17:**
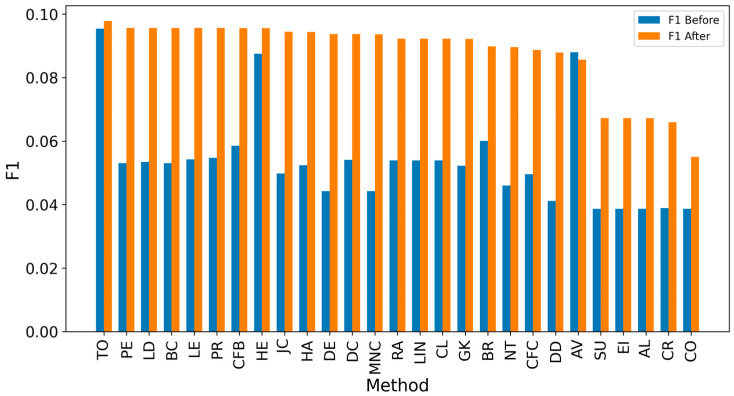
Average F1 score before and after expanding detected nodes by one hop for the Facebook network in the SIR-based simulation.

**Figure A18:**
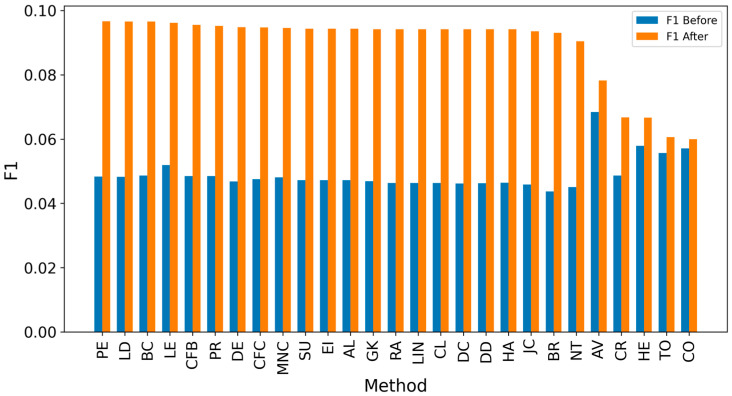
Average F1 score before and after expanding detected nodes by one hop for the Social network in the SIR-based simulation.

**Figure A19:**
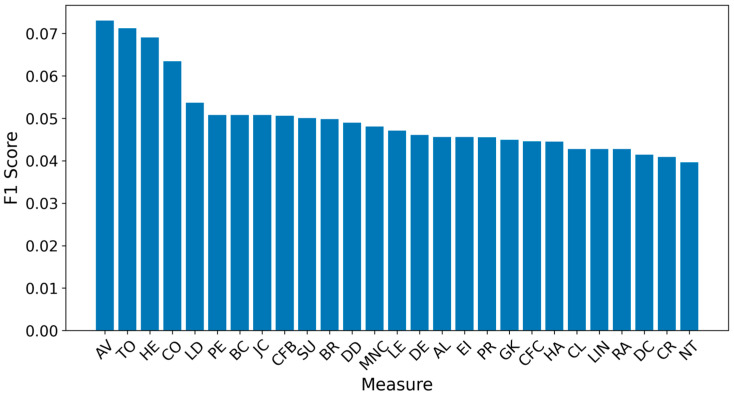
Global F1 score per measure in the IC-based simulation.

**Figure A20:**
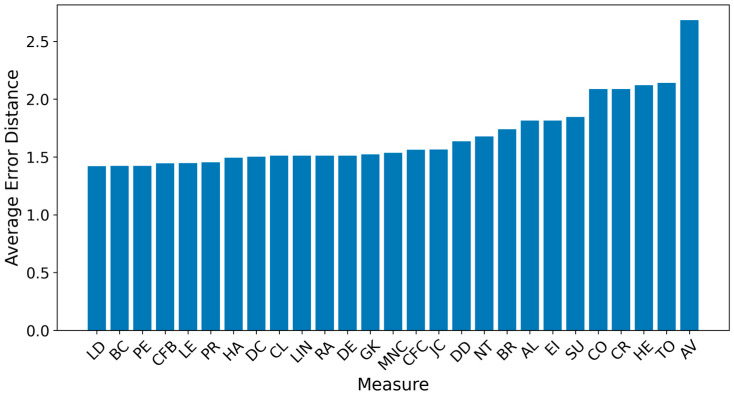
Global average distance per measure in the IC-based simulation.

**Figure A21:**
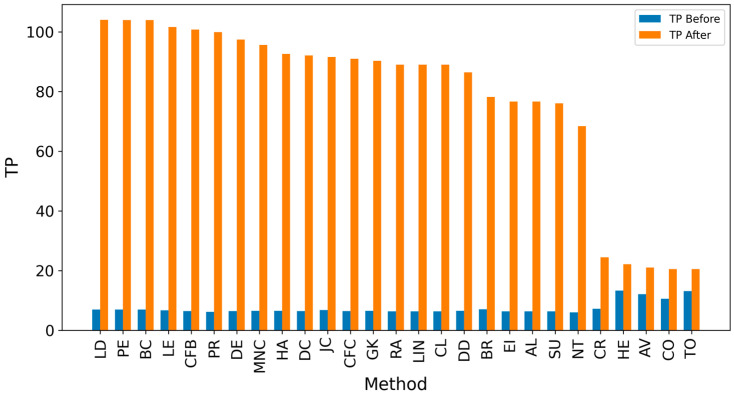
Average TP score before and after expanding detected nodes by one hop per method in the IC-based simulation.

**Figure A22:**
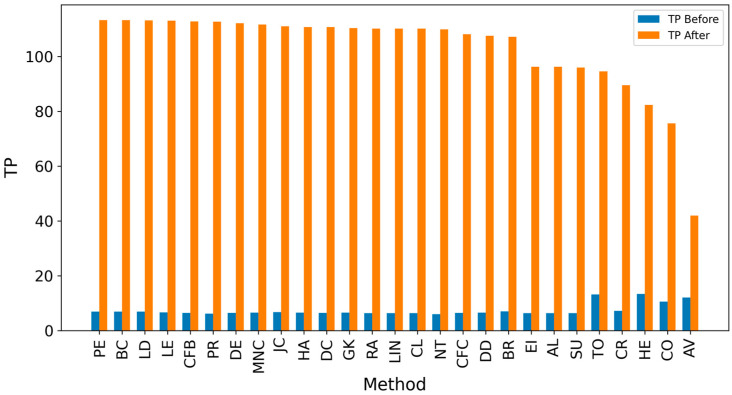
Average TP score before and after expanding detected nodes by two hops per method in the IC-based simulation.

**Figure A23:**
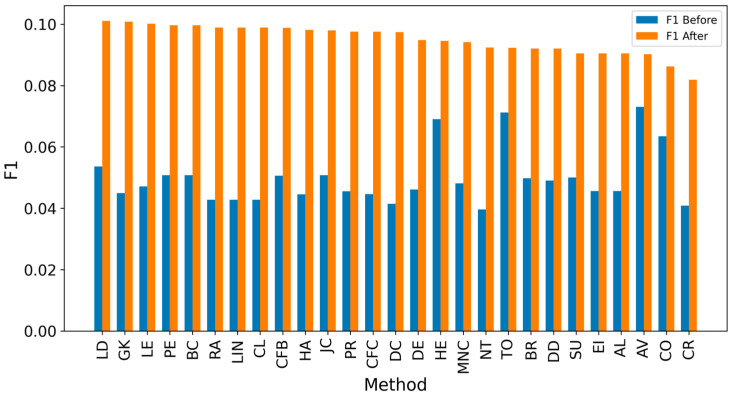
Average F1 score before and after expanding detected nodes by one hop per method in the IC-based simulation.

**Figure A24:**
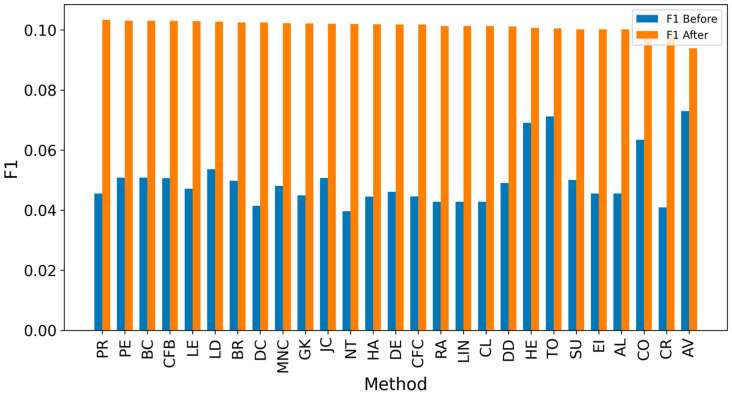
Average F1 score before and after expanding detected nodes by two hops per method in the IC-based simulation.

**Figure A25:**
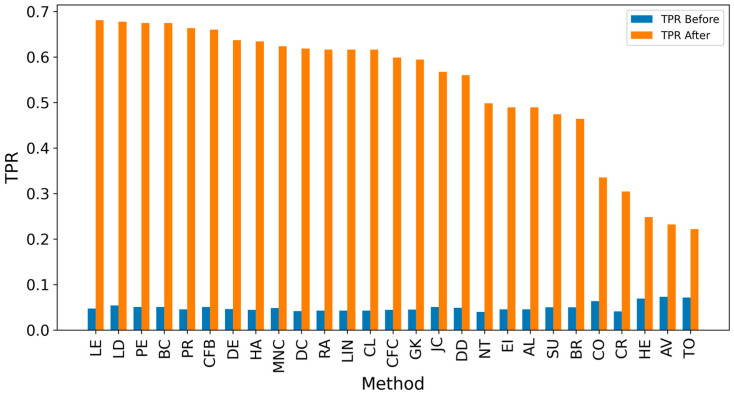
Average TPR score before and after expanding detected nodes by one hop per method in the IC-based simulation.

**Figure A26:**
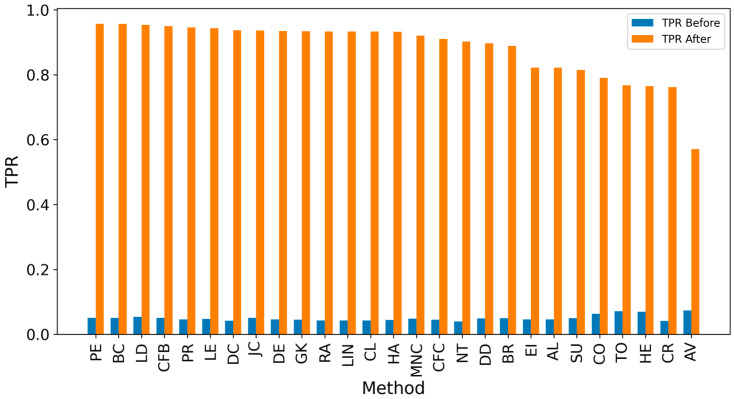
Average TPR score before and after expanding detected nodes by two hops per method in the IC-based simulation.

**Figure A27:**
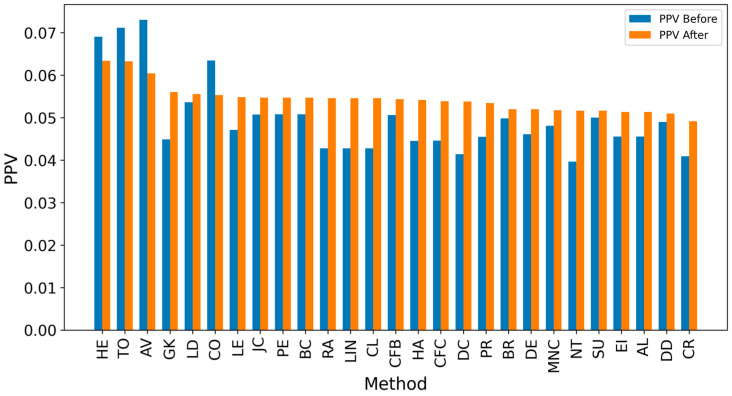
Average PPV score before and after expanding detected nodes by one hop per method in the IC-based simulation.

**Figure A28:**
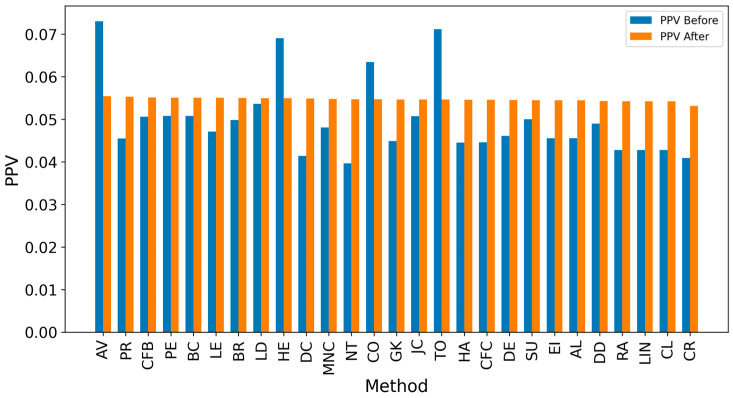
Average PPV score before and after expanding detected nodes by two hops per method in the IC-based simulation.

**Figure A29:**
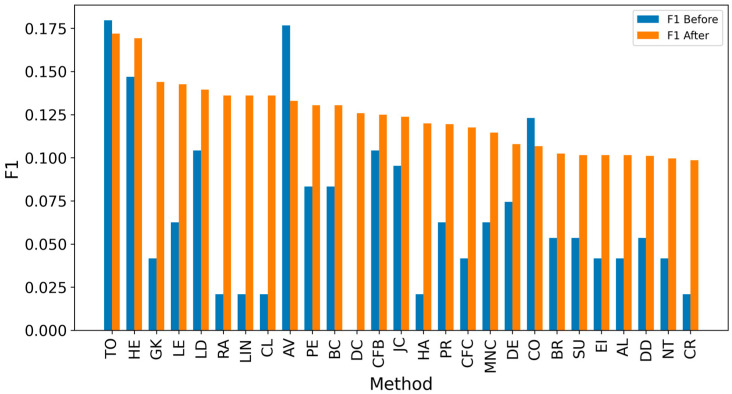
Average F1 score before and after expanding detected nodes by one hop for the Dolphin network in the IC-based simulation.

**Figure A30:**
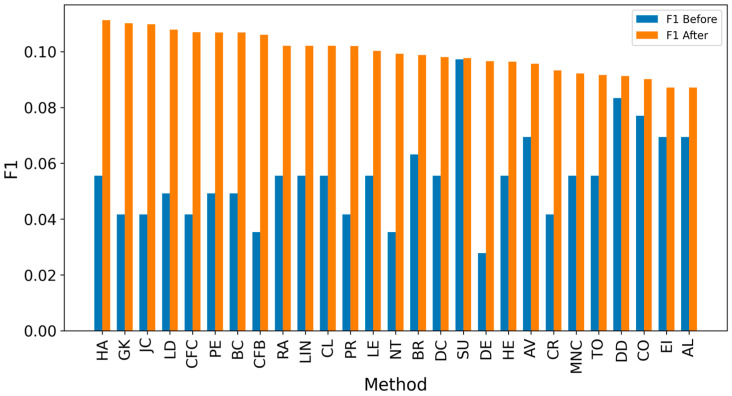
Average F1 score before and after expanding detected nodes by one hop for the Football network in the IC-based simulation.

**Figure A31:**
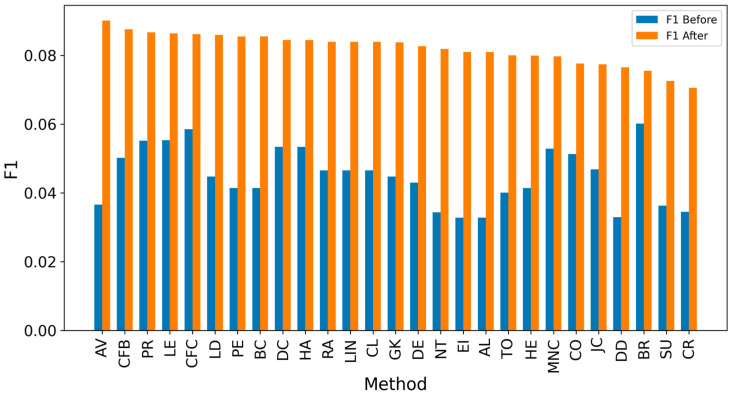
Average F1 score before and after expanding detected nodes by one hop for the SW-1 network in the IC-based simulation.

**Figure A32:**
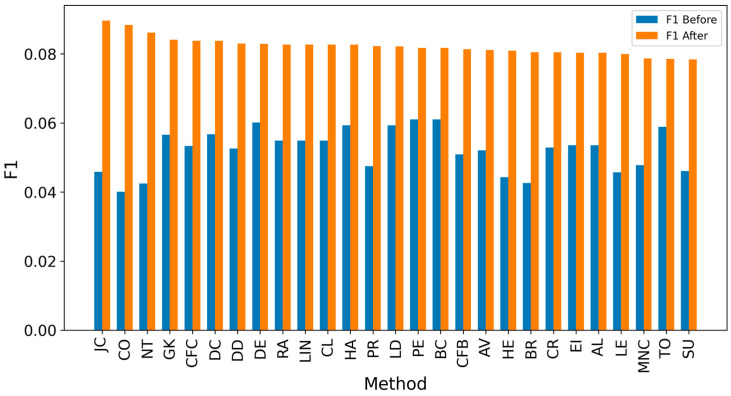
Average F1 score before and after expanding detected nodes by one hop for the SW-2 network in the IC-based simulation.

**Figure A33:**
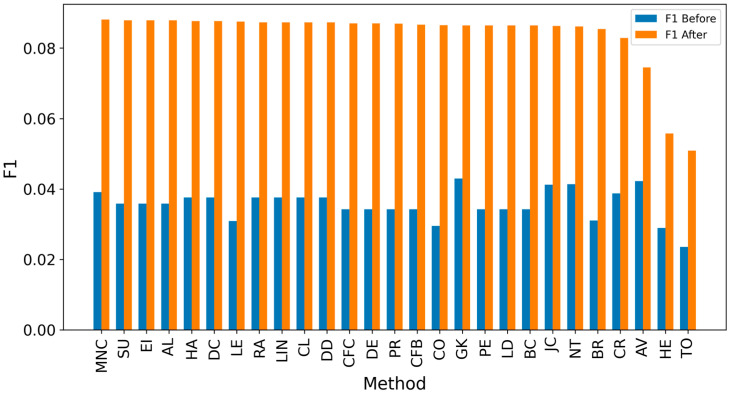
Average F1 score before and after expanding detected nodes by one hop for the SF-1 network in the IC-based simulation.

**Figure A34:**
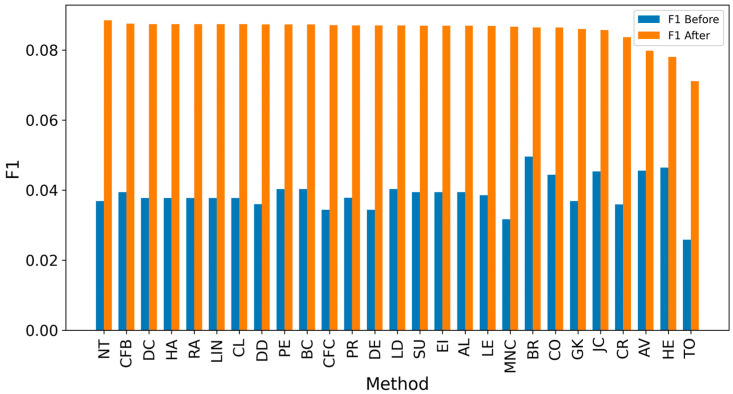
Average F1 score before and after expanding detected nodes by one hop for the SF-2 network in the IC-based simulation.

**Figure A35:**
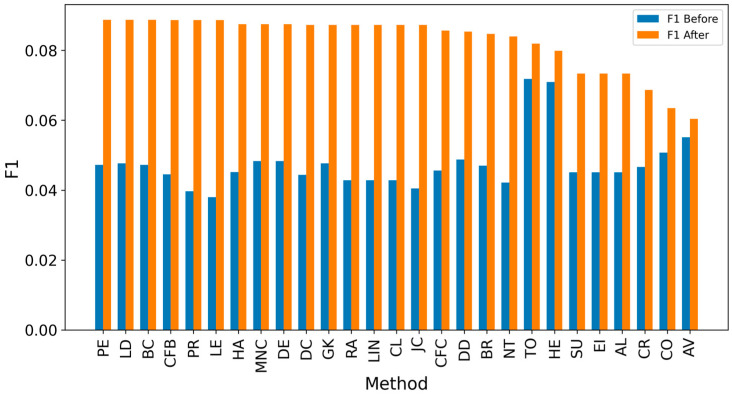
Average F1 score before and after expanding detected nodes by one hop for the Facebook network in the IC-based simulation.

**Figure A36:**
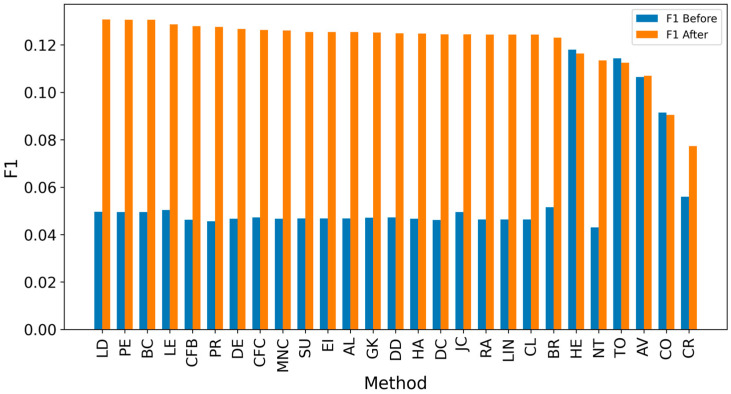
Average F1 score before and after expanding detected nodes by one hop for the Social network in the IC-based simulation.

## Data Availability

The data that support the findings of this study are available from the corresponding author upon reasonable request.
